# Wong-type dermatomyositis with interstitial lung disease and anti-SRP and -PM/Scl antibodies treated with intravenous immunoglobulin

**DOI:** 10.1016/j.jdcr.2023.08.016

**Published:** 2023-09-09

**Authors:** Eric H. Kowalski, H. Mark Kenney, Christopher T. Richardson

**Affiliations:** a Department of Dermatology, University of Rochester Medical Center, Rochester, New York; b University of Rochester School of Medicine and Dentistry, Rochester, New York; c Division of Allergy, Immunology and Rheumatology, University of Rochester Medical Center, Rochester, New York

**Keywords:** anti-PM/Scl antibody, anti-SRP antibody, dermatomyositis, intravenous immunoglobulin, interstitial lung disease, pityriasis rubra pilaris, Wong-type dermatomyositis

## Introduction

Dermatomyositis (DM) is an idiopathic inflammatory disorder with a heterogenous presentation. Patients exhibit varying degrees of concomitant cutaneous, muscle or other distinct extracutaneous manifestations that often allow for a clinical diagnosis. Wong-type DM is a rare variant with clinical and pathological overlap between DM and pityriasis rubra pilaris (PRP).^1^ The atypical clinical presentation of Wong-type DM often obscures and delays the diagnosis and workup. Herein, we present a case of refractory Wong-type DM with anti-signal recognition peptide (SRP) and anti-polymyositis/scleroderma (PM/Scl) antibodies, interstitial lung disease (ILD), and treatment response to intravenous immunoglobulin (IVIg).

## Case report

**Fig 1:**
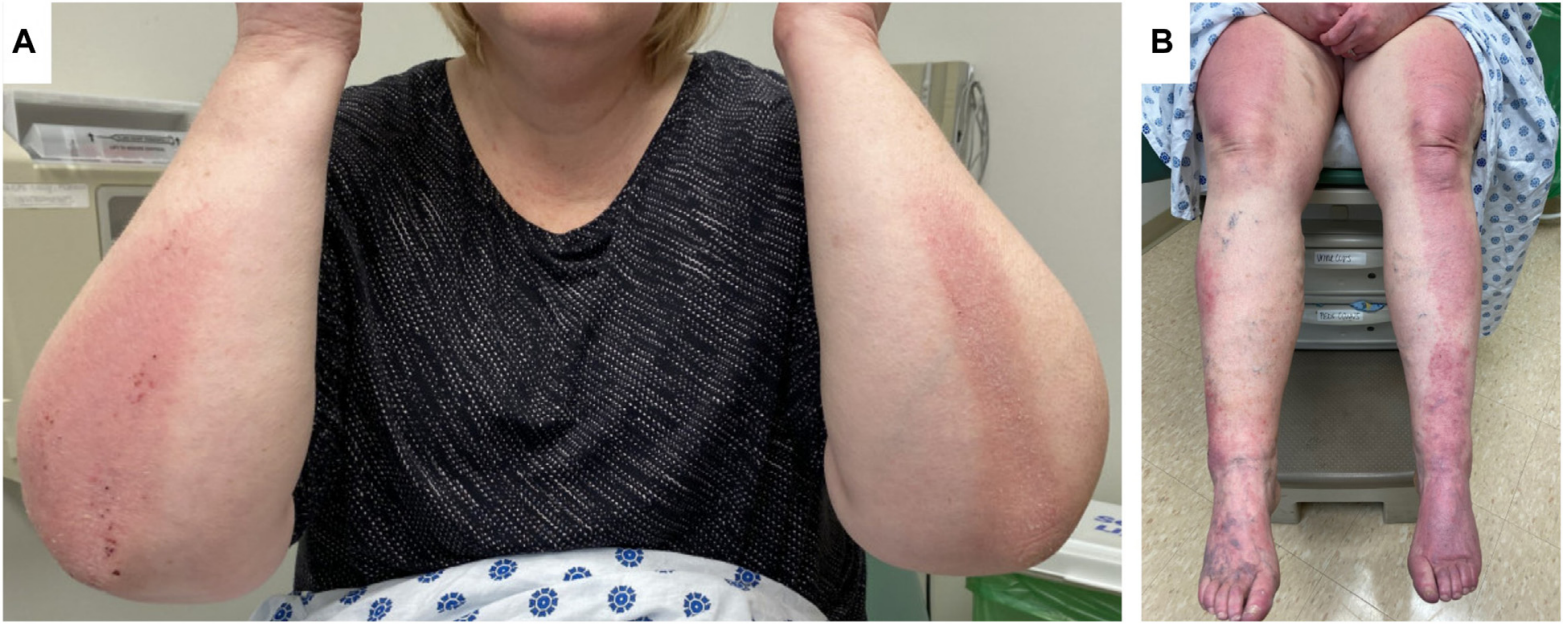
Psoriasiform plaques overlying the extensor surfaces of the upper (**A**) and lower (**B**) extremities.

A 52-year-old female with a history of poorly controlled psoriasiform plaques over her upper and lower extremities (Fig 1) presented for re-evaluation of her cutaneous disease following recent onset of severe ILD with positive antinuclear (1:320), SRP and PM/Scl-100 antibodies. Diagnosed with pathology confirmed PRP at age 12, she had failed numerous systemic immunomodulators and topical treatments including isotretinoin, methotrexate, cyclosporine, adalimumab, secukinumab, and most recently, ustekinumab. At initial presentation, she denied any muscle pain, weakness, dysphonia, or dyspnea.

**Fig 2:**
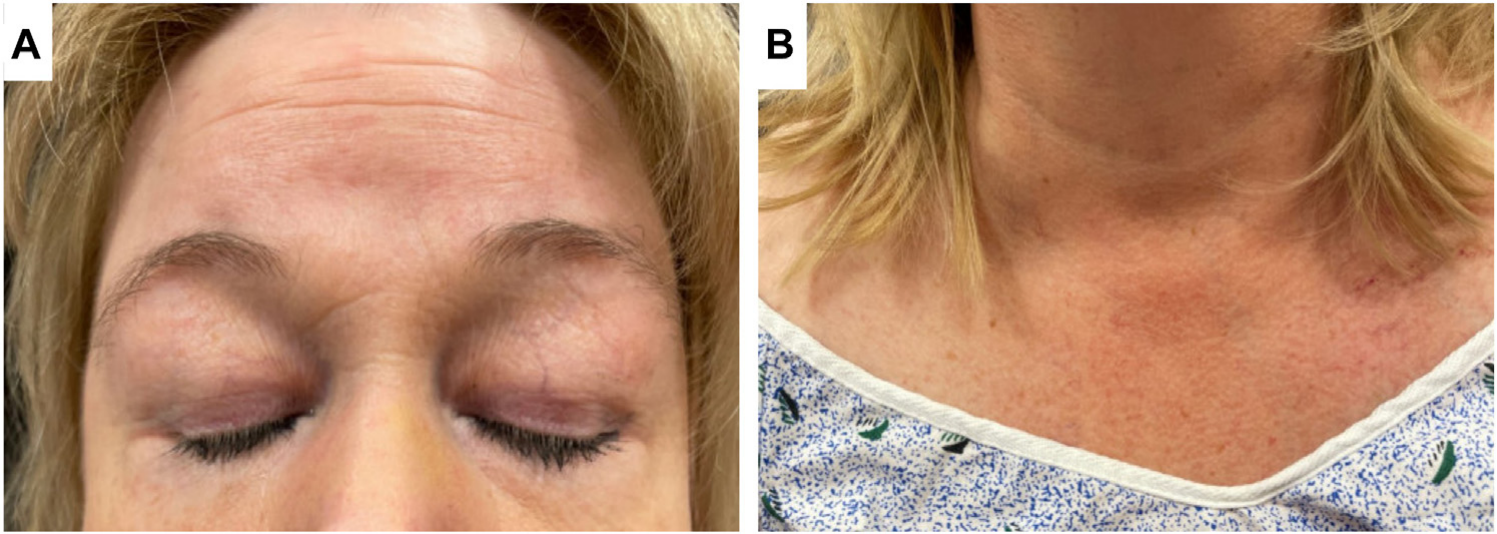
Cutaneous features of dermatomyositis, including heliotrope sign with violaceous erythema of the upper eyelids (**A**) and V-sign with erythema of the upper chest (**B**).

**Fig 3:**
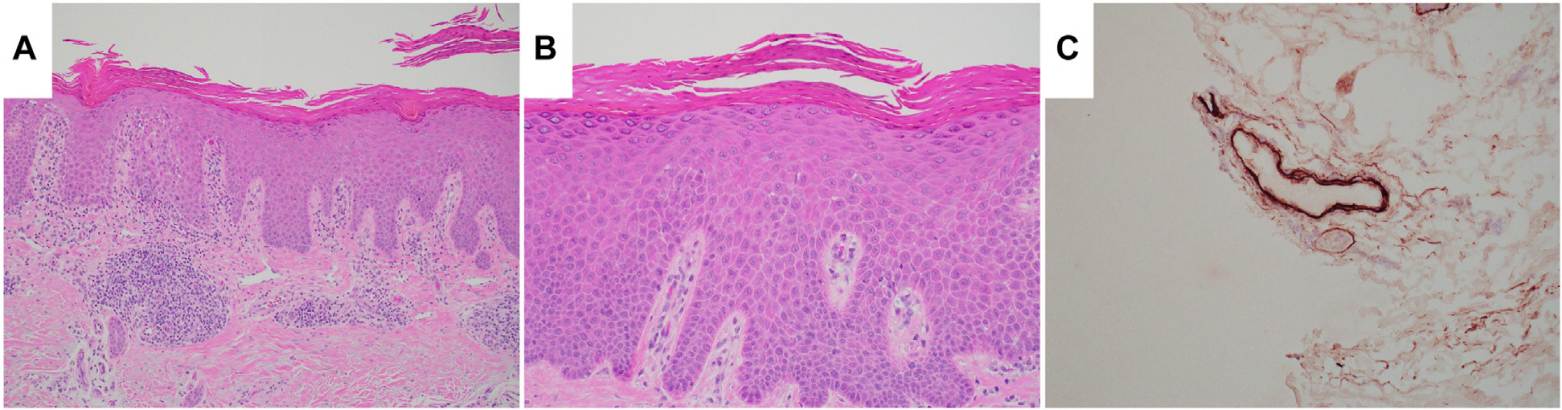
Hematoxylin and eosin (H&E) and immunohistochemistry (IHC) consistent with a diagnosis of Wong-Type Dermatomyositis. H&E staining demonstrates irregular psoriasiform hyperplasia, and lymphocytic infiltrate with scattered eosinophils in the papillary dermis and perivascular pattern (**A**) and foci of parakeratosis between orthokeratotic scale in a horizontal and vertical pattern (**B**). Intravascular capillary deposition of C5b-9 demonstrated by IHC (**C**). Original magnifications: (**A**) 20× (**B**) 40× (**C**) 40×.

Physical examination revealed psoriasiform plaques over the bilateral extensor forearms and elbows as well as the anterior, lateral, and posterior thighs. Subtle features consistent with DM were also observed, including heliotrope sign, erythema of the upper chest (Fig 2), and erythema over the knuckles. Skin biopsy revealed psoriasiform dermatitis with overlying confluent parakeratotic and orthokeratotic scale in a checkerboard pattern without epidermal atrophy or interface change, consistent with PRP (Fig 3, *A* and *B*). However, immunohistochemistry was positive for deposition of intravascular C3 and C5-9, while direct immunofluorescence was negative for IgA, IgG, and IgM, findings consistent with DM (Fig 3, *C*). Additional laboratory evaluation showed a low-titer positive cyclic citrullinated peptide along with normal creatine kinase and aldolase. This overlap of clinical, histologic and laboratory features of DM and PRP is consistent with Wong-type DM.

Given the severe and refractory disease, the patient was promptly initiated on monthly IVIg. Given concerns for muscle involvement, methylprednisolone was also initiated at 8 mg daily but quickly tapered to a maintenance dose of 4 mg daily. Several months later, the patient’s cutaneous disease was vastly improved; however, the patient reported dyspnea on exertion. Pulmonary function tests demonstrated a restrictive pattern consistent with ILD, which prompted initiation of mycophenolate mofetil.

## Discussion

Wong-type DM is a rare DM variant in which there is clinical and pathological overlap with PRP. Wong-type DM has been reported in children and adults with unclear consensus on whether this is an overlap syndrome of DM and PRP or a unique presentation of an already protean disease.^2^^,^^3^ Onset of PRP-like lesions varies and has been noted to occur prior to, simultaneously or after the diagnosis of DM. This variant often exhibits hyperkeratotic follicular papules with characteristic islands of sparing, as seen in PRP, but may present with classic cutaneous features of DM as well.^4^ Our case varies from typical Wong-type DM in that the primary PRP-like lesions are large psoriasiform plaques rather than scattered papules. The prominent linear distribution on the legs is also atypical for PRP.

The pathologic presentation classically reveals compact orthokeratosis alternating with parakeratosis in a checkerboard pattern as in PRP, though can include DM-features of vacuolar interface dermatitis or mucin deposition.^1^ Recently, findings of columnar dyskeratosis, defined as nonfollicular epidermal invaginations containing keratotic plugs with scattered dyskeratotic cells, has been postulated as a histological feature suggestive of Wong-type DM.^5^ To the best of our knowledge, this is the first reported case that demonstrates simultaneous findings of PRP on hematoxylin and eosin staining with concomitant immunohistochemistry findings consistent with DM. Unfortunately, we are unable to investigate the initial biopsies this patient received with her diagnosis in childhood. Ideally, this would delineate whether this was indeed Wong-type DM masquerading as PRP since childhood or if she developed DM superimposed on already present PRP.

As there are fewer than 45 cases of Wong-type DM in the literature, there remains no established connection with myositis-specific autoantibodies or risk of systemic involvement. Myositis-specific autoantibodies are associated with particular DM phenotypes and useful in prognostication of potential pulmonary involvement or association with a visceral malignancy. Our case expands the characterization of Wong-type DM to include anti-SRP and anti-PM/Scl antibodies in the setting of ILD. These autoantibody subtypes and ILD are both uncommon in juvenile-onset DM. Traditionally, anti-SRP and anti-PM/Scl have been associated with necrotizing myopathy and connective tissue disease overlap syndromes with ILD, respectively; these known relationships may provide valuable insights to guide extracutaneous workup in Wong-type DM patients. Further evaluation of myositis-specific autoantibodies in cases of PRP may also expedite diagnosis of unrecognized Wong-type DM. Additionally, although the prevalence of malignancy in Wong-type DM remains unclear,^4^ previous reports indicate that Wong-type DM may present as a paraneoplastic phenomenon, particularly with gynecological malignancies.^6^ Malignancy work-up in this case was negative.

Given the paucity of Wong-type DM, management and treatment remains anecdotal. Cutaneous DM has been traditionally treated first-line with various immunosuppressants. More recently, a seminal breakthrough in the treatment of DM was provided in the ProDERM trial, which investigated the use of IVIg in active DM patients. Although the primary clinical endpoint was reduction of myositis disease activity, there was clinically significant cutaneous improvement based on reductions in the Cutaneous DM Disease Area and Severity Index.^7^ Thus, IVIg provides an alternative treatment option for refractory or severe disease. Although the mechanism of action remains nebulous, IVIg is believed to play a multifaceted role in immunomodulation. To our knowledge, our case is the third report of refractory Wong-type DM being successfully treated with IVIg,^8^^,^^9^ and the first in the setting of ILD.^2^^,^^3^

This case draws attention to a rare clinical entity, Wong-type DM, that often masquerades as PRP. Although seldom encountered, maintaining Wong-type DM in a differential, especially when confronted with severely recalcitrant PRP associated with pulmonary symptoms, could allow for expedited diagnosis. Our case additionally highlights a unique presentation of Wong-type DM with anti-SRP and anti-PM/Scl antibodies associated with ILD and supports the therapeutic potential of IVIg in this rare condition.

## Conflict of interest

None disclosed.
